# Environmental, Lifestyle, Medical, and Dietary Factors Associated With Hie (Subjective Coldness) Among Japanese Women: A Web-Based Cross-Sectional Survey

**DOI:** 10.7759/cureus.104083

**Published:** 2026-02-22

**Authors:** Nozomu Mandai, Mayumi Watanabe, Takuya Nikaido, Tokimasa Takeda, Tsutomu Komine, Koshi Nakagawa, Chikako Tomiyama, Masae Ryufuku

**Affiliations:** 1 Faculty of Nursing Science, Tsuruga Nursing University, Tsuruga, JPN; 2 Faculty of Science and Engineering, Chuo University, Tokyo, JPN; 3 Department of Orthopaedic Surgery, Fukushima Medical University, Fukushima, JPN; 4 Preventive Medicine, Kyoto University, Kyoto, JPN; 5 Graduate School of Health Sciences, Niigata University, Niigata, JPN; 6 Preventive Medicine, Japan Biological Informatics Consortium, Tokyo, JPN

**Keywords:** analgesic use, dietary patterns, hie, japanese women, lifestyle habits, lower back pain, subjective coldness

## Abstract

Introduction

Hie (subjective coldness or sensitivity to cold) is a common complaint among Japanese women and has been discussed in relation to lower back pain and reduced well-being. Because subjective complaints often do not correspond to measured skin temperatures, environmental, lifestyle, medical, dietary, and psychosocial factors may contribute to the perception of Hie. This exploratory, hypothesis-generating study aimed to identify environmental, lifestyle, medical, and dietary factors associated with Hie status and symptom severity among Japanese women, with a focus on differences between mild (Hie (+)) and severe (Hie (++)) symptoms.

Methods

We conducted a web-based cross-sectional survey using an online Japanese research panel. Invitations were sent to 5,000 women selected through stratified random sampling by region; 1,000 women completed the survey. For the present analysis, we included only respondents who completed the entire survey and who indicated interest in the topic of Hie in a screening item, yielding a final sample of 341 women. Participants were categorized into three groups: (1) Hie (−): non-Hie group (n=162); (2) Hie (+): mild Hie group (n=114); and (3) Hie (++): severe Hie group (n=65), based on self-reported sensitivity to cold. Group differences were examined using chi-square tests or Fisher’s exact tests, as appropriate (two-sided p<0.05). The survey was conducted on January 20, 2022 (midwinter in Japan).

Results

Compared with the Hie (+) group, the Hie (++) group more often reported a present room temperature of <18°C (p=0.02) and used local/no heating rather than whole-room heating (p<0.01). Ongoing dietary restriction and nocturia ≥two times/night were also more common in the Hie (++) group (p<0.05). In contrast, the Hie (+) participants more commonly reported frequent intake of fish, beans, and fermented foods (p<0.05). Among analgesic users, the Hie (+) group more commonly obtained analgesics from drug stores/the Internet and used them mainly to manage menstruation symptoms, whereas the Hie (++) group more often obtained them from friends/family and used them for other pain/fever (Fisher’s exact tests, p<0.05).

Conclusion

Mild and severe Hie showed distinct association patterns. Severe Hie was associated with a present room temperature of <18°C, less whole-room heating, ongoing dietary restriction, and more frequent nocturia, whereas mild Hie was associated with higher intake of fish/beans/fermented foods and different self-medication patterns among analgesic users. Because this was a cross-sectional survey, causality cannot be inferred; prospective studies with objective measures and broader covariate assessment are warranted.

## Introduction

In traditional medicine, the subjective sensation of Hie (coldness) has been suggested as a potential contributing factor to lower back pain (LBP) [1]. Owing to its high prevalence among Japanese women, researchers have explored relationships between Hie and menstruation and other lifestyle factors, including diet [2-6]. Although reports indicate that 50% of female university students and 70% of female outpatients experience subjective coldness [4,7], its precise definition and factors associated with symptom severity remain unclear. Owing to its subjective nature, Hie is difficult to measure objectively. Furthermore, Kampo practitioners have discussed "hie-sho" as a subjective complaint and emphasize a holistic interpretation based on patients’ self-reported symptoms and constitutional patterns [8]. Kampo is a Japanese traditional medicine system practiced by licensed physicians in Japan and is often used alongside biomedicine. In Kampo-oriented care, hiesho (hie-sho) refers to a subjective coldness complaint and is typically addressed through individualized symptom assessment and lifestyle guidance to improve thermal comfort [8]. Physiological studies have explored objective correlates of cold constitution, including physical characteristics, living environment, vascular function, and skin temperature responses [9-11]. Together with evidence that severe Hie is associated with negative emotional states [12], these observations suggest that Hie may reflect a complex interaction between subjective perception and bodily states and that a multidisciplinary approach may be useful for its assessment and management.

Hie can reduce women’s well-being; however, factors associated with its presence and severity are not fully understood. In our previous cross-sectional study [12], severe Hie was associated with lower body weight/body mass index (BMI), chronic low back pain, and menstrual pain and was also related to negative emotional states. Because subjective coldness does not always correspond to objective temperature measures, understanding Hie likely requires consideration of environmental and behavioral contexts in addition to physiological factors.

To explore factors associated with Hie and more severe symptoms, a comprehensive approach is needed that considers not only thermal conditions but also lifestyle and health behaviors. Therefore, we collected information on the living environment, lifestyle habits, and medical/dietary factors among women who expressed interest in the topic of Hie.

This exploratory, hypothesis-generating study aimed to identify environmental, lifestyle, medical, and dietary factors associated with Hie status and symptom severity in a web-based cross-sectional survey of Japanese women, with a focus on factors distinguishing mild (Hie (+)) and severe (Hie (++)) symptoms.

## Materials and methods

Participants

This study used data from an online survey panel managed by a Japanese survey company (Cross Marketing Inc., Tokyo, Japan). Using stratified random sampling by region, invitations were emailed to 5,000 women registered in the panel (database of approximately 300,000 individuals). The survey company performed stratified sampling using pre-specified geographic region categories in its panel database to reduce regional imbalance. Of these, 1,000 women completed the survey and were included in the initial dataset. We included participants who answered all questions completely. The exclusion criterion was the use of specific medicines or supplements without a prescription (e.g., chemotherapeutic agents). The survey was conducted on January 20, 2022 (midwinter in Japan).

An online survey was administered to all participants using a questionnaire system provided by the survey company. Prior to study initiation, participants were informed that the data collected would be used for research purposes only and that strict confidentiality would be maintained. Informed consent was obtained from all participants before commencement of the study, and participants were informed of their right to withdraw at any time. This study was approved by the Medical Ethics Committee of Ibaraki Prefectural University of Health Sciences, Ibaraki, Japan (reference number e300-r120209).

In a screening item at the beginning of the questionnaire, respondents were asked whether they were personally interested in the topic of Hie (subjective coldness). For the present analysis, we retained only those who answered affirmatively to this screening item and provided complete responses (N=341). Participants in this analytic sample were aged 20-59 years. Accordingly, this analytic sample was not intended to estimate population prevalence of Hie by age group or region.

Survey questionnaire

The questionnaire was developed in Japanese to assess (1) self-reported Hie severity and (2) environmental, lifestyle, medical, and dietary factors (detailed information given in the sections below and full items in the Appendix). Item generation was informed by (a) items used in our prior Hie-related surveys (Watanabe et al., 2023) [13] and (b) relevant literature on cold sensitivity/Hie and indoor thermal environment (Takeuchi et al., 2008; World Health Organization, 2018; Umishio et al., 2020) [14-16]. In addition, we referred to qualitative comments from women experiencing Hie (approximately 10 individuals) to ensure that the item pool reflected commonly reported perceptions and coping behaviors in daily life. These steps were intended to support face and content validity for use in an epidemiologic survey setting.

Because a standardized, widely validated psychometric instrument for Hie severity has not been established for general web-based epidemiological surveys, Hie severity was assessed using a pragmatic single-item question with three response options (No/Yes (mild) / Yes (severe)), consistent with our prior work. Most covariates were measured as single items capturing concrete exposures or behaviors (e.g., room temperature category, heating type, smoking, nocturia frequency, and food intake frequency). Therefore, classical scale-based psychometric testing (e.g., internal consistency or factor analysis) was not applicable to the majority of items. The questionnaire has been used in our previous publications, supporting its feasibility for web-based data collection.

Grouping of participants

We divided participants into three groups: Hie (−), Hie (+), and Hie (++). Participants who selected “No” in response to the first question belonged to the Hie (−) group, those who answered “Yes (mild)” constituted the Hie (+) group, and the others constituted the Hie (++) group. Formal test-retest reliability was not assessed.

Environmental and lifestyle information

Participants were asked about their present room temperature (<18°C or ≥18°C), type of heating (whole room, local, or none), [17-19], winter-wear type (the same clothes worn throughout the year or warmer), and preferences for air conditioner and sunshine (dislike, N/A, or It is essential). 

Additionally, the following information was collected: frequency of cigarette smoking (never, occasionally, very often, or former smoker), self-reported exercise type (anaerobic, aerobic, or N/A (no exercise)), sitting duration in hours (less than one, one to two, two to four, or ≥four hours), sleep duration in hours [20-22] (less than six, six to seven, or ≥seven hours), frequency of nocturia (zero, one, two, or ≥three times), and bathing habits (shower only, short bath (<10 min), or long bath (≥10 min)). For statistical analysis, categories were collapsed to avoid small expected cell counts.

Former smokers were included in the “never” category (non-current smokers), and nocturia responses of two and ≥three were combined as ≥two times/night. Time since smoking cessation was not assessed.

Medical and dietary information

Information on the following variables was collected: dietary restrictions (ongoing, occasional, or none) and cold/flu status (no cold, before October, or after October). Additionally, the frequency of medical consultation for anemia treatment (currently under anemia treatment, history of anemia treatment, untreated anemia, a condition other than anemia, and “I have not seen a doctor”) and use of analgesics (twice a week (2/w), once a week (1/w), three times per month (3/m), occasionally, or never) were recorded. For statistical analysis, analgesic use frequency was recoded into three categories: 2/w, 1/w or 3/m (combined), and occasionally/no (combining occasionally and never) to avoid small expected cell counts.

We collected information from participants using analgesics, including how they obtained the medicine (doctor, drug store/Internet, or friend/family) and the reason for usage (headache, menstrual pain, back/knee pain, or skin disorder/fever). Analgesic users were defined as participants who reported any analgesic use (2/w, 1/w, 3/m, or occasionally); participants who answered “never” were treated as non-users and excluded from the analyses. Reasons for analgesic use were categorized as menstrual pain, back/knee pain, or skin disorder/fever; headache responses, if any, were grouped into the skin disorder/fever category.

In accordance with previous studies, we asked participants whether they had specific foods they liked or disliked and about their frequency of consumption of cold foods. We noted the participants’ consumption frequency of various foods, such as meat, fish, beans, and fermented food (frequently, N/A, or no) [7,23].

Statistical analysis

All statistical analyses were performed using IBM SPSS Statistics for Windows, Version 25 (Released 2017; IBM Corp., Armonk, New York, United States). Continuous variables were presented as mean ± standard deviation (SD) and categorical variables as frequencies and percentages. To examine factors associated with Hie status, we performed Pearson’s chi-square tests among the three groups (Hie (−), Hie (+), and Hie (++)). To explore factors associated with more severe Hie among women reporting Hie, we conducted chi-square tests between the Hie (+) and Hie (++) groups. A two-sided p-value <0.05 was considered statistically significant. When any expected cell count was small (less than five) in the Hie (+) versus Hie (++) comparisons, Fisher’s exact test was used (two-sided; Fisher-Freeman-Halton extension for r×c tables). Participants’ ages (categorized by decade) were also summarized for each group and compared using chi-square tests to assess basic background differences between groups.

## Results

Among the 341 respondents included in the present analysis, 162 were classified as Hie (−), 114 as Hie (+), and 65 as Hie (++). Tables 1, 2 summarize environmental/lifestyle and medical/dietary factors, respectively, and Table 3 summarizes analgesic acquisition and reasons among analgesic users.

**Table 1:** Environmental and lifestyle factors by Hie group (N=341) RT, room temperature; h, hours; N/A, not applicable. Values are n (% within each group). Present RT indicates the actual room temperature (°C) measured at the time of the survey (categories: <18°C or ≥18°C), not perceived room temperature. For analysis, former smokers were included in the 'never' category (non-current smokers; time since cessation not assessed), and nocturia responses of 2 and ≥3 were combined as ≥2. χ² values and p values are from Pearson’s chi-square tests. *p<0.05; **p<0.01.

Variable	Category	Hie (-) (n=162)	Hie (+) (n=114)	Hie (++) (n=65)	χ² (3 groups)	p value (3 groups)	χ² (Hie (+) vs Hie (++))	p value (Hie (+) vs Hie (++))
Environmental Information	Present RT (°C)				5.94	0.05	5.83	0.02*
	<18	61 (37.7%)	37 (32.5%)	33 (50.8%)				
	≥18	101 (62.3%)	77 (67.5%)	32 (49.2%)				
	Heating type				12.87	0.01*	10.51	<0.01**
	Whole room	103 (63.6%)	85 (74.6%)	33 (50.8%)				
	Local	47 (29.0%)	19 (16.7%)	22 (33.8%)				
	None	12 (7.4%)	10 (8.8%)	10 (15.4%)				
	Winter wear				59.51	<0.01**	0.01	0.94
	As usual	112 (69.1%)	31 (27.2%)	18 (27.7%)				
	Warmer	50 (30.9%)	83 (72.8%)	47 (72.3%)				
	Air conditioner preference				13.27	0.01*	2.45	0.29
	Dislike	42 (25.9%)	48 (42.1%)	28 (43.1%)				
	N/A	79 (48.8%)	36 (31.6%)	26 (40.0%)				
	It is essential	41 (25.3%)	30 (26.3%)	11 (16.9%)				
	Sunshine preference				14.85	<0.01**	3.81	0.15
	Dislike	35 (21.6%)	31 (27.2%)	22 (33.8%)				
	N/A	77 (47.5%)	31 (27.2%)	23 (35.4%)				
	It is essential	50 (30.9%)	52 (45.6%)	20 (30.8%)				
Lifestyle Information	Cigarettes				6.47	0.17	4.42	0.11
	Never	125 (77.2%)	87 (76.3%)	43 (66.2%)				
	Occasionally	18 (11.1%)	19 (16.7%)	11 (16.9%)				
	Very often	19 (11.7%)	8 (7.0%)	11 (16.9%)				
	Type of exercise				10.24	0.04*	2.60	0.27
	Anaerobic	31 (19.1%)	23 (20.2%)	12 (18.5%)				
	Aerobic	80 (49.4%)	45 (39.5%)	19 (29.2%)				
	N/A	51 (31.5%)	46 (40.4%)	34 (52.3%)				
	Sitting (h)				9.70	0.14	4.96	0.18
	<1	51 (31.5%)	21 (18.4%)	21 (32.3%)				
	1–2	35 (21.6%)	22 (19.3%)	11 (16.9%)				
	2–4	26 (16.0%)	27 (23.7%)	15 (23.1%)				
	≥4	50 (30.9%)	44 (38.6%)	18 (27.7%)				
	Sleep (h)				1.43	0.84	0.80	0.67
	<6	59 (36.4%)	44 (38.6%)	23 (35.4%)				
	6–7	46 (28.4%)	37 (32.5%)	19 (29.2%)				
	≥7	57 (35.2%)	33 (28.9%)	23 (35.4%)				
	Frequency of nocturia				16.17	<0.01**	7.19	0.03*
	0	110 (67.9%)	61 (53.5%)	35 (53.8%)				
	1	39 (24.1%)	43 (37.7%)	16 (24.6%)				
	≥2	13 (8.0%)	10 (8.8%)	14 (21.5%)				
	Bath				3.17	0.53	0.31	0.86
	Shower only	42 (25.9%)	36 (31.6%)	18 (27.7%)				
	Bath (<10 min)	47 (29.0%)	24 (21.1%)	14 (21.5%)				
	Bath (≥10 min)	73 (45.1%)	54 (47.4%)	33 (50.8%)				

**Table 2:** Medical and dietary factors by Hie group (N=341) 2/w, twice per week; 1/w, once per week; 3/m, three times per month; N/A, not applicable. Values are n (% within each group). Analgesic use frequency was recorded as 2/w, 1/w or 3/m (combined), and occasionally/no (occasionally + never) to avoid small expected cell counts. χ² values and p values are from Pearson’s chi-square tests unless otherwise indicated. For anemia (Hie (+) vs Hie (++)), Fisher’s exact test (Freeman-Halton extension) was used because of small expected cell counts (χ² not applicable). Because of multiple comparisons, interpret p values cautiously. *p<0.05; **p<0.01.

Variable	Category	Hie (-) (n=162)	Hie (+) (n=114)	Hie (++) (n=65)	χ² (3 groups)	p value (3 groups)	χ² (Hie (+) vs Hie (++))	p value (Hie (+) vs Hie (++))
Medical Information	Dietary restrictions				18.97	<0.01**	12.54	<0.01**
	Ongoing	40 (24.7%)	33 (28.9%)	27 (41.5%)				
	Occasionally	54 (33.3%)	53 (46.5%)	13 (20.0%)				
	None	68 (42.0%)	28 (24.6%)	25 (38.5%)				
	Cold/flu status				16.75	<0.01**	2.29	0.32
	No cold	137 (84.6%)	81 (71.1%)	42 (64.6%)				
	Before last Oct	21 (13.0%)	25 (21.9%)	14 (21.5%)				
	After last Oct	4 (2.5%)	8 (7.0%)	9 (13.8%)				
	Anemia				14.03	0.08	Fisher	0.16
	Anemia treatment (now)	3 (1.9%)	4 (3.5%)	0 (0.0%)				
	Anemia treatment (past)	9 (5.6%)	16 (14.0%)	3 (4.6%)				
	Anemia (no treatment)	20 (12.3%)	9 (7.9%)	5 (7.7%)				
	Other than anemia	8 (4.9%)	9 (7.9%)	6 (9.2%)				
	Have not seen a doctor	122 (75.3%)	76 (66.7%)	51 (78.5%)				
	Analgesics use				21.26	<0.01**	4.30	0.12
	2/w	5 (3.1%)	12 (10.5%)	2 (3.1%)				
	1/w or 3/m (combined)	20 (12.3%)	32 (28.1%)	15 (23.1%)				
	Occasionally/no	137 (84.6%)	70 (61.4%)	48 (73.8%)				
Dietary Information	Food likes or dislikes				11.75	0.02*	2.36	0.31
	Yes	24 (14.8%)	28 (24.6%)	17 (26.2%)				
	N/A	68 (42.0%)	28 (24.6%)	22 (33.8%)				
	No	70 (43.2%)	58 (50.9%)	26 (40.0%)				
	Cold foods				20.61	<0.01**	6.10	0.05
	Frequently	62 (38.3%)	66 (57.9%)	27 (41.5%)				
	N/A	57 (35.2%)	14 (12.3%)	16 (24.6%)				
	No	43 (26.5%)	34 (29.8%)	22 (33.8%)				
	Meat				10.28	0.04*	5.24	0.07
	Frequently	85 (52.5%)	80 (70.2%)	35 (53.8%)				
	N/A	53 (32.7%)	23 (20.2%)	18 (27.7%)				
	No	24 (14.8%)	11 (9.6%)	12 (18.5%)				
	Fish				19.70	<0.01**	6.92	0.03*
	Frequently	65 (40.1%)	76 (66.7%)	31 (47.7%)				
	N/A	60 (37.0%)	21 (18.4%)	22 (33.8%)				
	No	37 (22.8%)	17 (14.9%)	12 (18.5%)				
	Beans				24.32	<0.01**	10.58	<0.01**
	Frequently	68 (42.0%)	81 (71.1%)	31 (47.7%)				
	N/A	61 (37.7%)	23 (20.2%)	20 (30.8%)				
	No	33 (20.4%)	10 (8.8%)	14 (21.5%)				
	Fermented foods				19.59	<0.01**	6.89	0.03*
	Frequently	72 (44.4%)	80 (70.2%)	33 (50.8%)				
	N/A	59 (36.4%)	26 (22.8%)	23 (35.4%)				
	No	31 (19.1%)	8 (7.0%)	9 (13.8%)				

**Table 3:** Analgesic acquisition and reasons among analgesic users (N=149) Values are n (% within each group). P values from Fisher’s exact tests (Fisher–Freeman–Halton extension) among analgesic users due to small expected cell counts. Analgesic users were defined as participants reporting any analgesic use (2/w, 1/w, 3/m, or occasionally); those answering “never” were excluded. Headache responses, if any, were grouped into the skin disorder/fever category. *p<0.05, **p<0.01.

Variable	Category	Hie (−) (n=55)	Hie (+) (n=69)	Hie (++) (n=25)	P value (3 groups)	P value (Hie (+) vs. Hie (++))
Manner of obtaining					0.01*	<0.01**
	Doctor prescription	17 (30.9%)	15 (21.7%)	7 (28.0%)		
	Drug store/internet	32 (58.2%)	53 (76.8%)	13 (52.0%)		
	Friend/family	6 (10.9%)	1 (1.4%)	5 (20.0%)		
Reasons for use					0.02*	0.03*
	Menstrual pain	51 (92.7%)	61 (88.4%)	16 (64.0%)		
	Back/knee pain	1 (1.8%)	4 (5.8%)	4 (16.0%)		
	Skin disorder/fever	3 (5.5%)	4 (5.8%)	5 (20.0%)		

Below, we focus on the comparison between Hie (+) and Hie (++), which was used to explore factors associated with more severe symptoms among women reporting Hie. Participants were aged 20-59 years. Mean age was 42.86 years in Hie (−), 40.36 years in Hie (+), and 39.22 years in Hie (++). The age distribution of the participants by decade (20s/30s/40s/50s) was n=32/n=34/n=45/n=51 in Hie (−), n=25/n=29/n=44/n=16 in Hie (+), and n=18/n=19/n=14/n=14 in Hie (++) groups, respectively. The age distribution across the decades (20s, 30s, 40s, and 50s) did not differ significantly between Hie (+) and Hie (++) groups (chi-square test, p=0.12).

In the environmental domain (Table 1), present room temperature differed between Hie (+) and Hie (++): 50.8% of Hie (++) participants reported <18°C compared with 32.5% of Hie (+) participants (p=0.02). Heating type also differed (p<0.01); whole room heating was more common in Hie (+) (74.6%) than in Hie (++) (50.8%), whereas local/no heating was more common in Hie (++) (49.2%) than in Hie (+) (25.5%). Differences in winter wear, air conditioner preference, and sunshine preference were not statistically significant between Hie (+) and Hie (++) (all p>0.05).

In the lifestyle domain (Table 1), nocturia was more frequent in Hie (++): the proportion reporting nocturia ≥two times/night was 21.5% in Hie (++) versus 8.8% in Hie (+) (p=0.03). Smoking status, exercise type, sitting duration, sleep duration, and bathing habits did not differ significantly between the two groups (all p>0.05).

In medical information (Table 2), dietary restriction patterns differed between Hie (+) and Hie (++) (p<0.01). Ongoing dietary restriction was more common in Hie (++) (41.5%) than in Hie (+) (28.9%), whereas occasional dietary restriction was more common in Hie (+) (46.5%) than in Hie (++) (20.0%). No significant differences were observed for cold/flu status, anemia status, or overall frequency of analgesic use between the two groups (all p>0.05).

In dietary intake (Table 2), Hie (+) participants more frequently reported frequent intake of fish (66.7% vs. 47.7%, p=0.03), beans (71.1% vs. 47.7%, p<0.01), and fermented foods (70.2% vs. 50.8%, p=0.03) compared with Hie (++). The frequency of cold food intake was higher in Hie (+) than in Hie (++) (57.9% vs. 41.5%) and reached borderline significance (p=0.05). Food likes/dislikes and meat intake did not differ significantly between groups (p>0.05).

Analgesic use frequency differed among the three groups (overall p<0.01), but not between Hie (+) and Hie (++) (p=0.12) (Table 2). Therefore, we examined analgesic users in more detail (Table 3; N=149). The manner of obtaining analgesics differed between Hie (+) and Hie (++) (Fisher’s exact p=0.004): Drug store/Internet was more common in Hie (+) (76.8%) than in Hie (++) (52.0%), whereas obtaining analgesics from friends/family was more common in Hie (++) (20.0%) than in Hie (+) (1.4%). Reasons for use also differed (Fisher’s exact p=0.025); use for menstrual pain was more common in Hie (+) (88.4%) than in Hie (++) (64.0%), while use for back/knee pain or skin disorder/fever was more common in Hie (++). 

## Discussion

In this web-based survey of women interested in the topic of Hie, we observed that the Hie (+) (mild) and Hie (++) (severe) groups differed in several environmental, medical/dietary, and behavioral patterns. In two-group comparisons, severe Hie was associated with a present room temperature of <18°C and less whole-room heating, a higher prevalence of ongoing dietary restriction, and more frequent nocturia. In contrast, mild Hie was associated with more frequent intake of fish, beans, and fermented foods and distinct patterns of analgesic acquisition and use among analgesic users. These findings suggest that mild and severe Hie may not simply represent sequential stages along a single pathway but may reflect different clusters of associated factors (Table 4).

**Table 4:** Difference between Hie (+) and Hie (++) groups across domains RT, room temperature. Summary of significant differences between Hie (+) and Hie (++) based on the comparisons between the groups in Tables 1-3.

Domain	Hie (+)	Hie (++)
Environmental factors	Present RT (≥18°C); Whole room heating	Present RT (<18°C); Local/no heating
Dietary restriction	Occasional	Ongoing
Nocturia (≥2 times/night)	8.8%	21.5%
Dietary pattern	Fish/beans/fermented foods; more frequent	Fish/beans/fermented foods; less frequent
Analgesic pattern (users)	Drug store/Internet; Menstruation	Friend/family; Other pain/fever

The environmental differences may reflect both thermal exposure and coping strategies. Compared with Hie (+), women in the Hie (++) group more often reported a present room temperature of <18°C and relied more on local or no heating. Although directionality cannot be determined, indoor thermal conditions and heating practices may be linked to perceived coldness severity and may represent a target for individualized lifestyle counseling (e.g., maintaining adequate indoor warmth in winter).

Dietary restriction showed the clearest medical difference between groups. Ongoing dietary restriction was more common in Hie (++), whereas occasional restriction was more common in Hie (+). Because the survey was cross-sectional, these associations may reflect different motivations (e.g., weight-control efforts, health conditions, or responses to symptoms) rather than a causal effect of dieting on Hie severity. In addition, Hie (+) participants reported more frequent intake of fish, beans, and fermented foods. This dietary pattern may indicate greater engagement with health-related information or dietary practices among women with mild symptoms, but further research is needed to confirm this interpretation.

Nocturia (≥two times/night) was more frequent in the Hie (++) group. Nocturia may be related to sleep disruption, urinary symptoms, or other underlying conditions, none of which were assessed in detail in this questionnaire. Future studies should incorporate more detailed clinical and psychosocial measures to clarify why nocturia co-occurs with severe Hie.

Overall frequency of analgesic use did not differ significantly between Hie (+) and Hie (++); however, among analgesic users, acquisition routes and reasons for use differed. The Hie (+) group more often obtained analgesics from drug stores/the Internet and used them primarily for menstrual pain, whereas the Hie (++) group more often obtained them from friends/family and used them for other pain or fever. These patterns may reflect differences in self-medication behavior and symptom profiles and should be explored in future work.

Clinical implications

The observed patterns highlight several practical domains for clinical history-taking among women reporting Hie/hiesho, including indoor thermal conditions and heating practices during winter, dietary restriction behaviors, nocturia, and analgesic acquisition/use patterns. These factors may help clinicians (including Kampo physicians) tailor individualized lifestyle counseling and identify co-occurring symptoms that warrant further evaluation, while recognizing that causality cannot be inferred from this cross-sectional survey.

Patient implications

For patients experiencing Hie, attention to potentially modifiable domains, such as maintaining adequate indoor warmth in winter and reviewing ongoing dietary restriction, and seeking assessment for nocturia or persistent pain symptoms may be relevant considerations. Prospective studies incorporating objective measures and broader covariate assessment are needed to confirm these hypothesis-generating observations.

Several factors frequently discussed in the Hie literature, such as body weight/BMI and emotional states (e.g., anger) and related autonomic responses, were not measured in the present survey. In our previous study [12], severe Hie was associated with lower BMI and negative emotional states; integrating such measures alongside environmental and behavioral factors may help build a more comprehensive model of Hie in future longitudinal studies.

Limitations

This study has limitations. First, the sample was restricted to respondents who expressed interest in the topic of Hie, which may limit generalizability and introduce selection bias. Therefore, these results should not be interpreted as population prevalence estimates (including age-stratified prevalence). Second, all variables were self-reported and assessed at a single time point; therefore, causal relationships cannot be inferred. Third, multiple comparisons were performed, and findings should be interpreted cautiously. Despite these limitations, the present results highlight potentially actionable domains - indoor thermal practices, dietary restriction, nocturia, diet, and self-medication patterns - that may inform hypothesis generation and individualized counseling. In addition, because the survey was conducted in midwinter (January 20, 2022), associations related to heating practices and present room temperature may not generalize to other seasons. Moreover, although the age distribution did not differ significantly between Hie (+) and Hie (++), we did not perform multivariable adjustment; residual confounding by unmeasured factors remains possible. Accordingly, findings should be interpreted as patterns of association rather than causal effects. In addition, objective physiological measures (e.g., skin temperature) and socioeconomic and mental health variables were not assessed, which may contribute to residual confounding.

An additional limitation is that the questionnaire was not a formally psychometrically validated scale for Hie; rather, it was a pragmatic, study-specific set of items developed from prior surveys and literature. Measurement error or misclassification of Hie severity and related behaviors is therefore possible.

## Conclusions

In this web-based cross-sectional survey of Japanese women who expressed interest in Hie, mild and severe Hie showed distinct patterns of associated environmental, lifestyle, and medical/dietary factors. Severe Hie was associated with a present room temperature of <18°C, less whole room heating, ongoing dietary restriction, and more frequent nocturia, whereas mild Hie was associated with higher intake of fish/beans/fermented foods and different analgesic self-medication patterns among analgesic users. 

These findings suggest that Hie severity may reflect different clusters of behavioral and environmental contexts rather than a simple linear progression of symptoms. Although causality cannot be inferred from this cross-sectional design, the identified domains-indoor thermal environment, dietary practices, nocturia, and analgesic use patterns-may provide practical points for individualized lifestyle counseling and clinical assessment. Prospective studies incorporating objective measures and multivariable adjustment are warranted.

## Appendices

Questionnaire

Q Do you suffer from Hie? If yes, is it mild or severe?

□ No   □ Yes (mild)   □ Yes (severe)

Warmth Information

Q Present room temperature (RT ℃)

□ <18°C   □ ≥18°C

Q Which type of heating do you use?

□whole room   □local   □none

Q Your winter wear is..

□ the same clothes that I wear throughout the year □ warmer   

Q Do you like air conditioning?

□ dislike □ N/A □ It is essential

Q Do you like sunshine?

□ dislike □ N/A □ It is essential

Lifestyle Information

Q Frequency of cigarette smoking

□ never   □ occasionally   □ very often   □ former smoker

Q Type of exercise

□ anaerobic   □ aerobic   □ N/A

Q How long do you sit in a day? (duration of sitting hours)

□ <1   □ 1-2   □ 2-4   □ ≥4

Q How long do you sleep (hours) per day?

□ <6    □ 6-7   □ ≥7

Q How often do you go to the bathroom during sleep at night? (Frequency of nocturia)

□ 0   □ 1   □ 2   □   ≥3

Q What is your bathing style?

□ shower only   □ bath (<10 min)   □ bath (≥10 min)

Medical Information

Q Dietary limitations

□ ongoing   □ occasionally   □ no

Q Have you had a cold?

□ no □ yes, before last October (- Oct)   □ Yes, after last October(Oct-)

Q Have you visited a doctor for any of the following reasons?

□ undergoing anemia treatment □ past anemia treatment □ untreated anemia

□ conditions other than anemia □ I have not visited the doctor for any of these reasons

Q How often do you use analgesics?

□ twice per week (2/w)  □ once per week  (1/w)  □ thrice per month (3/m)  □ occasionally  □ never

Dietary Information

Q Do you have food preferences or food aversions?

□ yes   □ N/A   □ no

Q How often do you consume cold foods?

Q How often do you consume meat?

Q How often do you consume fish?

Q How often do you consume beans?

Q How often do you consume fermented foods?

□ frequently   □ N/A   □ no

Analgesics Information

The following two questions are only for participants who indicated using analgesics in the previous question.

Q If you use analgesics, indicate how you obtain them

□ doctor   □ drug store/internet   □ friend/family

Q If you use analgesics, indicate the reason for usage

□ headache   □ menstrual pain   □ back/knee pain   □ skin disorder/fever
